# Gendered health institutions: examining the organization of health services and men’s use of HIV testing in Malawi

**DOI:** 10.1002/jia2.25517

**Published:** 2020-06-26

**Authors:** Kathryn Dovel, Shari L Dworkin, Morna Cornell, Thomas J. Coates, Sara Yeatman

**Affiliations:** ^1^ Division of Infectious Diseases David Geffen School of Medicine at UCLA Los Angeles CA USA; ^2^ Partners in Hope LilongweAngeles Malawi; ^3^ School of Nursing and Health Studies University of Washington Bothell Bothell WA USA; ^4^ Centre for Infectious Disease Epidemiology & Research School of Public Health & Family Medicine University of Cape Town Cape Town South Africa; ^5^ Department of Health and Behavioral Sciences University of Colorado Denver Denver CO USA

**Keywords:** gender health disparities, health institution, HIV, HIV testing, men, sub‐Saharan Africa

## Abstract

**Introduction:**

Men in sub‐Saharan Africa are less likely to use HIV testing services than their female counterparts. Norms of masculinity are frequently cited as the main barrier to men’s use of HIV testing services, but very little is known about how health institutions are organized to facilitate or impede men’s care. We examined the organization of health institutions in Malawi, and implications for men’s use of HIV testing services.

**Methods:**

A mixed methods ethnography was conducted in Malawi between October 2013 and September 2014. National Ministry of Health guidelines from 2012 to 2014 were analysed, counting the frequency of recommended preventative services by sex. In‐depth interviews were conducted with 18 healthcare workers and 11 national key informants (29 total). Five rural health facilities participated in direct observation and 52 observational journals were completed to document the structure and implementation of HIV services within local facilities. All data were analysed using the theory of gendered organization. Findings were grouped into one of the three theoretical levels of organization: (1) organizational policy; (2) organizational practice; and (3) structure of gendered expectations.

**Results:**

Health institutions were gendered across three levels. *Organizational policy*: National guidelines omitted young and adult men’s health during reproductive years (176‐433 recommended visits for women vs. 32 visits for men). Health education strategies focused on reproductive and child health services, with little education strategies targeting men. *Organizational practice*: HIV testing was primarily offered during reproductive and child health services and located near female‐focused departments within health facilities. As these departments were women’s spaces, others could easily tell that men were using HIV services. *Structure of gendered expectations*: Clients who successfully accessed HIV testing services were perceived as exemplifying characteristics that were traditionally considered feminine: compliance (obeying instructions without explanation); deference (respecting providers regardless of provider behaviour); and patience (“waiting like a woman”).

**Conclusions:**

Health institutions in Malawi were organized in ways that created substantial, multilevel barriers to men’s HIV testing and reinforced perceptions of absent, difficult men. Future research should prioritize a gendered organization framework to understand and address the complex realities of men’s constrained access to HIV services.

## 1 INTRODUCTION

Men in sub‐Saharan Africa are less likely than women to test for HIV, initiate treatment and remain in antiretroviral therapy programmes [1]. Men’s absence from HIV testing services is particularly concerning given that HIV testing is the entry point for all other HIV services [2]. Across eight countries in sub‐Saharan Africa, 22% of men living with HIV remain undiagnosed compared to 10% of women[3]. In recent years, extensive efforts have been made to better engage men [4], however, gender disparities in HIV testing likely still remain [3]. Importantly, men are also underrepresented in other health services, such as tuberculosis [5]. In light of the persistent gender disparities in HIV and other health services, there is an urgent need to identify institutional barriers to men’s use of services, and interventions to resolve them.

The structure of health institutions and *how* health services are offered matters. Men are less likely to use HIV testing services when providers are perceived as rude [6, 7], testing services are not private [8], services are offered at inconvenient hours [9], or require long travel or wait times  [8]. The organization of HIV programmes at the national and international level may also contribute to men’s underrepresentation in care. In an analysis of 146 World Health Organization global or regional HIV guidelines, women were three‐times more likely to be mentioned than men (13,882 vs. 4, 302)  [10]. Men’s representation improved slightly when excluding publications dedicated to children, women and PMTCT, however, even in “gender neutral” documents, women were still twice as likely to be mentioned as men (7,719 vs. 3,697 times). Similarly, among 119 policy documents from the President’s Emergency Plan for AIDS Relief, men were rarely mentioned, and when they were, men were often portrayed as perpetrators of the epidemic  [11]. Men’s absence from international guidelines is important because such documents largely shape national policies, and in turn, the structure of HIV services in local health facilities [12, 13]. Donor guidelines and priorities are especially influential in countries that rely heavily on donors to fund national programmes. For example, in 2016 only 14% of the Malawi HIV response was funded domestically [14]. 

To date, extensive work has been done describing barriers to men's access to services both on the supply and demand side, but gaps remain. On the supplyside, research has focused on narrow components of health institutions, such as patient–provider interactions or the structure of individual services [9]. There is limited knowledge about *how* health institutions are gendered across multiple levels of health policy, practice and patient–provider interactions. On the demand side, narrow and constraining norms of masculinity, characterized by physical strength, aggression, sexual performance and sexual risk taking, have been shown to limit men’s use of HIV and other health services [13, 15]. However, most research has focused on how harmful gender norms influence healthcare seeking behaviour among individual men. Little is known about how these same gender norms are embedded within, and perpetuated through, the organization of health institutions.

Health institutions, like other social organizations, are shaped by dominant social norms and social inequalities [16]. The few studies that have explored how health institutions are organized have found that men in various regions had fewer opportunities than women to engage with health institutions, and with HIV testing services more specifically [17, 18, 19, 20]. We build on this work by examining the gendered organization of health institutions across multiple levels in Malawi, and whether institutions are structured to facilitate health services utilization for one gender over the other.

We applied the theory of gendered organization that asserts that organizations are built on and reproduce not only gender norms, but also gender inequalities [16]. The theory has been predominantly applied to women’s entry and advancement in traditionally male occupations [21, 22]. The theory posits that organizations shape and reaffirm harmful gender norms across three levels (examples given from literature on women in the workplace): (1) *organizational policy*, including the absence of maternity leave, breastfeeding accommodations and protection against sexual harassment [23]; (2) *organizational practice*, such as impromptu high‐level meetings after work hours [24]; and (3) the *structure of gendered expectations*, such as when traditionally male characteristics are required for success within the organization, for example commitment to full‐time or intensive careers over family obligations [25, 26].

We examined how Malawi’s health institutions are organized, and the implications of gendered health institutions on men’s use of HIV testing services.

## 2 METHODS

### 2.1 Study design

We analysed mixed methods data from an ethnographic study conducted in Malawi between October 2013 and September 2015. The full protocol is described elsewhere [12]. The study included: national health policy documents; in‐depth interviews with providers and key informants; and observations of HIV services offered within five health facilities in Zomba District, Malawi. Facilities within Zomba varied in size and location, ranging from a large central hospital to smaller rural facilities. The district had an 11% HIV prevalence at the time of the study [27] and men comprised 35% of all adult HIV testers [28].

### 2.2 Data collection

#### 2.2.1 National policy

We analysed national health policy and guideline documents  published between 2010 to 2015 from the Malawi Ministry of Health (n=6) [29, 30, 31, 32, 33, 34]. We included national guidelines for broader health services (not just HIV services) because these can influence how men and women engage with health institutions, and provide key opportunities for HIV education and provider‐initiated HIV testing [13]. National policies and recommendations can inform how local health facilities are organized and how men and women understand their role in the health system [12]. Across all policies, we summarized preventative health services recommended for men and women of reproductive age (15‐44 years), capturing the age groups with the highest risk of HIV infection for both men and women [27].

#### 2.2.2 In‐depth interviews

Four research assistants conducted 29 in‐depth interviews: 18 with healthcare providers (including the chief or “in‐charge” for each facility and providers who offered HIV testing services) and 11 with national‐level key informants (HIV policy makers within the Ministry, HIV programme managers and local HIV researchers). Interview guides focused on: (1) implementation of HIV testing policies; (2) perception of men as clients; and (3) perception of barriers to men’s use of HIV testing services. Oral consent was attained from all respondents. Interviewers and respondents were matched by gender. Interviews lasted approximately 45 minutes and were conducted in either Chichewa or English. All interviews were recorded, transcribed and translated into English (if necessary).

#### 2.2.3 Observational journals

Observational journals were conducted by the same four research assistants at five health facilities in Zomba, Malawi. Observational journals are a form of local ethnography used to examine the social dimensions of HIV in sub‐Saharan Africa [28, 29, 30]. Observational Journals are well suited to capture practices within a health institution: documenting what people say and how they interact with one another within the health facility, which can be different from what they report in traditional research settings [29]. While observational journals cannot capture all events within one setting, they provide an important overview of common interactions and conversations within routine settings [29].

Research assistants took detailed field notes about observed interactions. At the end of each day, field notes were compiled into detailed observational journals, describing the content and context of interactions. Observational journals documented: (1) location of HIV testing services within the health facility, as well as services offered near HIV testing; (2) times when HIV testing was offered; (3) composition of clients in the waiting spaces near HIV testing services and (4) interactions between clients, and between clients and providers within waiting spaces, including clinic protocols observed. HIV testing consultations were not observed. Research assistants captured general interactions within the facility, and not only barriers for men. Observational journals were conducted over a five‐month period, with research assistants spending a consecutive 4 to 8 weeks observing each facility on a full‐time basis. Fifty‐two observational journals (20‐typed pages each) were completed in English. Descriptions of the activities were consistent across research assistants, providing confidence in their accuracy.

### 2.3 Data analysis

For national guidelines, we used simple count techniques to count the number of health visits required annually and cumulatively (from 15 to 44 years) for men and women to meet national preventative health service recommendations [38]. For in‐depth interviews and observational journals, we developed a codebook using deductive and inductive techniques [39]. Data were first coded by the level of gendered organization: (1) organizational policy; (2) organizational practice; and (3) the structure of gendered expectations. Within each level, we developed deductive codes based on existing literature. Inductive codes were added as transcripts were analysed. The codebook was finalized after reading 10 in‐depth interview transcripts and five observational journals. Qualitative data were analysed using Atlas.ti [40] and were triangulated to develop a coherent, holistic description of findings [41].

### 2.4 Ethics approvals

Ethical approval was received from the Colorado Multiple Institutional Review Board and Malawi’s National Health Sciences Review Committee.

## 3 RESULTS

Below we examine the organization of health services across the three levels of the gendered organization (Table 1). We also explore implications of a gendered health institution on men’s use of HIV testing services.

**Table 1:** Summary of the gendered health institution for HIV testing services in Malawi, by level of the gendered organization

Level of gendered organization	Example in Malawi
Organizational policy	
Health service recommendations	Malawi guidelines recommend *176‐433* health service during women's reproductive lifespan (15‐44 years) versus *32* health service for men in the same period
Health education	Health education services were not provided at locations or health services frequented by men
Organizational practice	
Service availability	HIV testing primarily available during hours of ANC services
Physical environment	HIV testing often located near or in ANC, family planning or under‐five services
Structure of gendered expectations	
Successful clients have feminine characteristics	Successful clients exemplify feminine characteristics: patience (waiting); compliance (obeying instructions without explanation); and deference (respecting providers)

ANC, antenatal care.

### 3.1 Organizational policy

#### 3.1.1 National recommendations for health services

Table 2 describes Malawi guidelines for health services and the cumulative number of health visits required across one’s reproductive lifetime (15‐44 years) to meet recommendations.

**Table 2:** Malawi Ministry of Health recommended health services and estimated visits required across the reproductive lifespan (15‐44 years)

Service	Target population	Estimated number of visits between 15‐44 years			
		Women: five‐year FP (implant; 9%^a^)	Women: quarterly FP (injectables; 23%^a^)	Women: monthly FP (pills; 2%^a^)	Men
ANC	Women	18	18	18	**–**
Delivery	Women	4	4	4	**–**
Post‐natal	Women	4	4	4	**–**
Family planning	Women	7	88	264	**–**
Under‐five	Women	120	120	120	**–**
HIV testing	Women and men	23	23	23	29
Circumcision	Men	**–**	**–**	**–**	3
Total		176	257	433	32

ANC, antenatal care; FP, family planning.

^a^ DHS 2016[27]

Five of the seven recommendations were for women’s reproductive health or children under five years of age. Most recommendations required multiple visits each year. High fertility rates in Malawi (4.4 children) [27] meant that women were expected to attend 176‐433 reproductive or child health visits during their reproductive lifetime; an average of 6‐16 visits/year.

Recommendations for men of the same age range included annual HIV testing and voluntary medical male circumcision (VMMC), resulting in 32 health service visits during men’s reproductive lifetime; or an average one visit per year. Estimate justifications and assumptions are provided in Table S1.

Key informants believed that men’s absence from national recommendations had several important consequences. Men did not have universal entry points for HIV testing and therefore few opportunities for provider‐initiated‐testing‐and‐counselling (PITC), a critical strategy to increase testing coverage at a population level.

Clinics do not have favourable conditions for men to go for HIV testing … Men can only come to the clinic for HIV testing. That’s all. But women can go [anytime] … Women go when the child is born and when the child is sick, the child is tested and the mother as well. And women must also test during pregnancy. But there is no service to encourage men to come to the hospital where they can also be tested. (Male provider, Facility 5)

#### 3.1.2 Health education

Men’s limited engagement with health institutions also meant they had little limited exposure to health education. Further, most health education strategies were offered alongside female‐focused services, such as antenatal care (ANC), family planning, and underfive services, and in the morning hours. Key informants believed limited exposure to health education contributed to men’s poor knowledge and awareness of health services.

Men are certainly unequipped [to use HIV services]. Men are not given skills. Since they were born up they are ignored about health and about taking care of families. They are completely left alone because people assume they already know. So what do we expect from men? They have no knowledge. Men run away from the clinic. … We think that shows power? No, men are scared … Women are at the clinic multiple times and are given information. Men are not. (Male key informant, local researcher)

In contrast, key informants believed women were frequently exposed to HIV services and educational materials. One female provider suggested that ANC services gave women a “mind for testing” because they learned how to overcome barriers to testing and normalized testing for women at key milestones in their lifetime (Female provider, Facility 4).

Some key informants reported that the lack of recommendations for men’s health led men to reject health institutions altogether because they believed they had no place within them.

All programmes are targeting women. They [men] say, “Fine. We will move the other way.” And by the time we [health institution] decide that we want to address men, we now have to chase them. It’s obvious that we haven’t been fair to women as far as rights are concerned. But now to try to solve the problem we are creating another problem. (Male key informant, HIV District Coordinator)

### 3.2 Organizational practice

#### 3.2.1 Service availability

In large hospitals (two of five facilities), HIV testing services were available during most facility hours. In small, rural health centres (three of five facilities), HIV testing was primarily offered alongside ANC services, usually in the early morning and on specific days. Testing could be accessed outside these hours, but often required extended wait times before a provider was available. In four of five facilities, the study team observed non‐pregnant clients turned away from HIV testing because a qualified provider was not available.

Here there is no one [provider] who is specialized for HIV testing … we have someone, but after antenatal [they] are sometimes busy with other things. People wait and get tired so they choose to go. (Male Provider, Facility 2)

Limited service availability may affect both men and non‐pregnant women. However, women’s universal entry point during ANC services means that most women will be able to access HIV testing throughout their lifetime, regardless of challenges related to service availability during non‐ANC health visits.

#### 3.2.2 Physical environment

Testing services were often located near ANC and/or children under‐five services (three of five facilities). Providers reported that the only reason for men to be in these traditionally female‐oriented spaces were involvement in ANC (which was rare) or HIV services. Since clients often waited many hours to receive care, providers acknowledged that men faced an increased risk of being identified as accessing HIV services.

If a woman walks into this building people can think of those [under‐five and antenatal services]. But a man, if a man comes to this building people definitely think, ‘Ah, HIV’. (Male provider, Facility 3)

### 3.3 Structure of gendered expectations

Observational journals and interviews with providers revealed three key traits clients needed to successfully access HIV testing services: (1) compliance; (2) deference; and (3) patience, all characteristics traditionally viewed as feminine. Findings from in‐depth interviews were similar across both male and female providers.

Clients were expected to *comply* with provider instructions, often without a description of *why* the instructions were given. Nearly all providers mentioned that, in practice, clients were rarely given explicit reasons as to why specific tests, treatments or follow‐up appointments were recommended. One provider explained, “You just say, ‘HIV testing is important. You should go get tested tomorrow or whenever you can’. That’s it.” (Female provider, Facility 5).

Clients who were *deferential* were perceived to have better experiences within the facility. Clients were expected to accept rude behaviour from providers. Those who questioned providers’ authority were often shouted at or refused services. Clients who were willing to accept rude behaviour from providers may have been more likely to continue attending health facilities, even after negative experiences, and therefore more likely to be exposed to HIV testing services in the future.

Successful clients were viewed as *patient* and waited extended hours to receive services. While facilities had set working hours, the availability of facility staff varied widely, with frequent staff absences. Furthermore, the number of clients seeking care were often extremely high, so that even when providers were present, clients still faced extended wait times of two or more hours. Most providers recognized that men did not like waiting with women. Clients would criticize men who waited in long queues, mocking men for “waiting like a woman.” A female research assistant observed:

Two young men stood on a line of women. One of the [women] said that “males do not stand in line. They are wasting their time there as if they are females.” … (Female Research Assistant, Facility 4).

## 4 DISCUSSION

We examined the gendered organization of health institutions and its influence on men’s use of HIV testing services in Malawi. Taking an institutional, multi‐level approach, we examined holistic barriers within the Malawi health institutions that may deter men’s use of not only HIV services, but health services more broadly. We found that the health institutions were organized around children’s and women’s reproductive health in ways that limited or discouraged men’s engagement in care, leading to substantial, multi‐level barriers to men’s HIV testing within health institutions. While we examined the implications of gendered health institutions on men’s use of HIV testing services, these same institutional barriers likely impact men’s representation in other health services as well.

Within *organizational policy*, national health guidelines provided few recommendations for men of reproductive years (15‐44 years) to access routine care as compared with women of the same age range, resulting in minimal routine health visits for men. Universal entry points for men were limited to HIV testing, VMMC and acute care. Lack of entry points meant there were few natural opportunities to routinely offer PITC to men, a critical strategy for HIV testing [42]. The lack of a universal entry points for men may further perpetuate community‐level socialization that health services and health facilities are women’s spaces [7, 43]. Routine outpatient services could provide an entry point for men. However, we found that general health education was not common within these services, suggesting a missed opportunity to offer HIV testing and referral for treatment. Men’s limited engagement with health institutions contributed to what key informants described as major gaps in men’s knowledge and familiarity with health institutions. Other research in the region has previously described limited health literacy and knowledge among men [4].

Within *organizational practice*, HIV testing services were not readily available except during times when ANC, family planning or under‐five services were being offered. This resulted in longer than usual wait times for outpatient attendees, and at times, complete unavailability of HIV testing. Furthermore, HIV services were often offered in or near women’s reproductive and children’s health departments, making it difficult for men to access confidential care. Our findings support previous reports that the feminized environment of facilities may lead to unwanted HIV status disclosure for men [44].

Within the *structure of gendered expectations*, successful clients needed to have characteristics that were considered traditionally “feminine". These included (1) compliance (obeying provider instructions without an explanation as to *why* instructions were given), (2) deference (being respectful and passive to providers, regardless of provider behaviour), and (3) patience (waiting extended hours). Other studies also describe the ideal (or successful) client as self‐motivated, compliant, passive and deferential [45, 46, 47]. Providers may be more likely to refuse or provide sub‐optimal care to men who do not demonstrate these ideal characteristics. In Zimbabwe, for instance, providers were less likely to offer HIV testing to children accompanied by male versus female guardians [48]; and in Uganda, providers excluded some men during antenatal visits, even if men attended as part of a male involvement programme [49].

What can be done to address gendered health institutions in the context of HIV services? One strategy widely implemented across sub‐Saharan Africa is outreach services for men. HIV self‐testing, community, index and work‐based HIV testing strategies generally increase testing among men [9]. However, outreach strategies alone will not alleviate health disparities for men. For HIV‐positive men, sustained engagement in health facilities after testing positive is critical for long‐term outcomes.

Immediate strategies are needed to address organizational structures that contribute to gendered health institutions and their negative health effects for men. Such changes promise to improve not only men’s use of HIV testing services, but men’s use of other services in which they have historically been underrepresented. First and foremost, universal entry points for men are essential. Potential entry points include outpatient departments [50], fatherhood and non‐communicable disease screening for older men [51]. Male‐focused health education services that resonate with men’s priorities and concerns have been found effective in some settings [4]. Improved infrastructure to facilitate private HIV testing spaces near outpatient departments, along with innovative strategies to reduce testing wait times such as facility‐based HIV self‐testing [52] or increased staffing, may be required. Finally, innovative strategies are needed to address negative male stereotypes held by policy makers and health care workers. To date there are few interventions aimed to change negative male stereotypes, and additional research is needed to identify specific strategies that can help to this end.

### 4.1 Limitations

This study has several limitations. First, data are from 2013 to 2015 and may not reflect the current situation on the ground. However, recent literature show that men are still absent from national guidelines [53] and continue to comprise the minority of HIV services [1], suggesting that our findings are still relevant. Second, observational journals cannot capture all events within participating health facilities, and may exclude mundane events that research assistants did not notice or deemed unworthy of documentation. Additional limitations of observational journals are discussed in depth elsewhere [36]. Third, social desirability bias is common in in‐depth interviews and cannot be excluded as a possibility within the current study. Fourth, this paper does not examine men’s interpretation and internalization of gendered organizations or the gendered interactions within health institutions. This deserves further exploration in future research. Finally, findings may not be generalizable outside Malawi,  however, similar gendered health institutions likely exist throughout sub‐Saharan Africa as national HIV programmes are largely influenced by international donor guidelines and priorities.

## 5 CONCLUSIONS

The gendered health institution in Malawi created substantial, multi‐level barriers to men’s use of HIV testing services that straddle organizational policy, organizational practice and the gendered expectations of how clients should interact with providers. Future research should prioritize a gendered organization framework to address the complex reality of men’s engagement with HIV services.

## COMPETING INTERESTS

We declare no competing interests.

## AUTHORS’ CONTRIBUTIONS

KD conceived the study. KD and SY contributed to study design. KD developed the guideline and training. KD analysed the data. KD, SY and SD developed the theoretical framework. KD, SY, SD, MC and TC provided substantial scientific input into the interpretation of results. KD took the lead in drafting the manuscript. SY, SD, MC and TC provided substantial comments to improve the draft. All authors contributed to the collection or interpretation of data, provided critical revisions to the report and approved the final draft.

## FUNDING

Data collection was funded by the National Institute of Mental Health under Grant F31‐MH103078. KD’s time was partially supported by the National Institute of Mental Health under grant T32‐MH080634‐10 and Fogarty International Center K01‐TW011484‐01. KD receives support from the UCLA CFAR grant AI028697 and the UCLA AIDS Institute. SY’s time was partially supported by the National Institute of Child Health and Human Development under Grant R01‐HD077873. MC’s time was partially supported by the National Institutes of Health’s National Institute of Allergy & Infectious Diseases under Award Number U01AI069924; & the National Institute of Mental Health (NIMH) & the South African Medical Research Council (#R01MH106600) & the Fogarty International Center (FIC) under award number D43TW011308. The funders had no role in study design, data collection and analysis, decision to publish or preparation of the manuscript.

## Supporting information


**Table S1.** Justifications and assumptions for estimates described in Table 1: Malawi Ministry of Health recommended health services and estimated visits required across the reproductive lifespan (15‐44 years).
