# Supplementary material for: Gendered health institutions: examining the organization of health services and men’s use of HIV testing in Malawi
Source: J Int AIDS Soc. 2020 Jun 26;23(Suppl 2):e25517. doi: 10.1002/jia2.25517 (PMC7319160; doi:10.1002/jia2.25517)
Supplement: Supplementary file 1 — Table S1. Justifications and assumptions for estimates described in Table 1: Malawi Ministry of Health recommended health services and estimated visits required across the reproductive lifespan (15‐44 years). [file JIA2-23-e25517-s001.pdf]

Supl 1. Justifications and assumptions for estimates described in Table 1: Malawi Ministry of Health recommended health services and estimated visits required across the reproductive lifespan (15-44 years)

			Justification for estimated number of visits between 15-44 years			
Service	Frequency	Target population	Women: 5-year FP (Implant; 9%*)	Women: quarterly FG (Injectable; 23%*)	Women: monthly FP (Pills; 2%*)	Men
ANC	17.6	Women	4 ANC visits * 4.4 pregnancies = 18 visits	4 ANC visits * 4.4 pregnancies = 18 visits	4 ANC visits * 4.4 pregnancies = 18 visits	-
Delivery	4.4	Women	1 delivery visit * 4.4 pregnancies = 4 visits	1 delivery visit * 4.4 pregnancies = 4 visits	1 delivery visit * 4.4 pregnancies = 4 visits	-
Post-natal	4.4	Women	1 post-natal visit * 4.4 pregnancies = 4 visits	1 post-natal visit * 4.4 pregnancies = 4 visits	1 post-natal visit * 4.4 pregnancies = 4 visits	-
Family planning	7-264	Women	1 family planning visit/3 years * 22 years (assuming women start family planning at 17 years of age = 27 years of family planning - 5 years for pregnancy and/or trying to get pregnant) = 7 visits	4 family planning visits per year * 22 years (assuming women start family planning at 17 years of age = 27 years of reproductivity - 5 years for pregnancy and/or trying to get pregnant) = 88 visits	12 family planning visits per year * 22 years (assuming women start family planning at 17 years of age = 27 years of reproductivity - 5 years for pregnancy and/or trying to get pregnant) = 264 visits	-

Under five services	120	Women	12 under-five visits per year * 10 years - a rough estimate of how long women may have a child under 5 years of age = 120 visits	12 under-five visits per year * 10 years - a rough estimate of how long women may have a child under 5 years of age = 120 visits	12 under-five visits per year * 10 years - a rough estimate of how long women may have a child under 5 years of age = 120 visits	-
HIV testing	23-29	Women and men	1 HIV test per year * 22.6 years (years during pregnancy were not included since PMTCT testing is incorporated into ANC visits) = 23 visits	1 HIV test per year * 22.6 years (years during pregnancy were not included since PMTCT testing is incorporated into ANC visits) = 23 visits	1 HIV test per year * 22.6 years (years during pregnancy were not included since PMTCT testing is incorporated into ANC visits) = 23 visits	1 HIV test per year * 29 years = 29 visits
Circumcision	3	Men	-	-	-	3 visits for 1 VMMC procedures = 3 visits

* DHS 2016
